# Mass transport and charge transfer through an electrified interface between metallic lithium and solid-state electrolytes

**DOI:** 10.1038/s42004-023-00923-4

**Published:** 2023-06-15

**Authors:** Leon Katzenmeier, Manuel Gößwein, Leif Carstensen, Johannes Sterzinger, Michael Ederer, Peter Müller-Buschbaum, Alessio Gagliardi, Aliaksandr S. Bandarenka

**Affiliations:** 1 Technical University of Munich, TUM School of Natural Sciences, Department of Physics, Physics of Energy Conversion and Storage, James-Franck-Str. 1, 85748 Garching, Germany; 2 TUMint·Energy Research, Lichtenbergstr. 4, 85748 Garching bei München, Germany; 3 Technical University of Munich, TUM School of Computation, Information and Technology, Department of Electrical and Computer Engineering, Hans-Piloty-Straße 1, 85748 Garching bei München, Germany; 4 Technical University of Munich, TUM School of Natural Sciences, Department of Physics, Chair for Functional Materials, James-Franck-Str. 1, 85748 Garching, Germany; 5 Heinz Maier-Leibnitz Zentrum (MLZ), Technical University of Munich, Lichtenbergstr. 1, 85748 Garching, Germany

**Keywords:** Surfaces, interfaces and thin films, Batteries

## Abstract

All-solid-state Li-ion batteries are one of the most promising energy storage devices for future automotive applications as high energy density metallic Li anodes can be safely used. However, introducing solid-state electrolytes needs a better understanding of the forming electrified electrode/electrolyte interface to facilitate the charge and mass transport through it and design ever-high-performance batteries. This study investigates the interface between metallic lithium and solid-state electrolytes. Using spectroscopic ellipsometry, we detected the formation of the space charge depletion layers even in the presence of metallic Li. That is counterintuitive and has been a subject of intense debate in recent years. Using impedance measurements, we obtain key parameters characterizing these layers and, with the help of kinetic Monte Carlo simulations, construct a comprehensive model of the systems to gain insights into the mass transport and the underlying mechanisms of charge accumulation, which is crucial for developing high-performance solid-state batteries.

## Abstract (web summary)

All-solid-state lithium-ion batteries are promising energy storage devices owing to their safe use and high energy density, whereby understanding electrode and solid electrolyte interfaces is key for battery development. Here, the authors use spectroscopic ellipsometry, impedance measurements, as well as Monte Carlo simulations to elucidate the formation of charge depletion layers at the electrode/electrolyte interface.

## Introduction

All-solid-state batteries (ASSB) attract increasing attention as a promising alternative to traditional Li-ion batteries due to their potentially higher energy density, longer lifespan, and improved safety^1, 2^. The solid-state electrolyte (SSE) used in ASSBs replaces the liquid or polymer electrolyte used in conventional Li-ion batteries and enables the use of metallic lithium (Li(s)) anode^3,4^. The holy grail of the anode materials promises 3860 mAh g^−1^^5^, but it is inherently challenging to stabilize them due to the formation of dendrites and inhomogeneous plating/stripping reactions^6,7^. As such, one of the significant challenges in developing solid-state batteries is the charge accumulation at the Li(s)/SSE interface^8^. This charge accumulation occurs due to the mismatch in electrochemical potential between the Li(s) and the SSE, forming a space charge layer (SCL) at the interface^9,10^. The SCL can cause significant changes in the local concentration of mobile Li-ions in the SSE, leading to increased interfacial resistance^11,12^.

The concept of SCL, describing a depletion or accumulation of mobile Li-ions, has been the focus of our previous work; until now, however, only under ion-blocking conditions^13^. In this case, with no mass transport across any of the two interfaces, the Li-ions will deplete on one side and thus accumulate on the far side of the electrolyte^14^. Globally, charge neutrality prevails in the SSE^15^, with the total amount of additional charge at the two interfaces being equal. The blocking electrode configuration was previously studied using electrochemical impedance spectroscopy (EIS) and spectroscopic ellipsometry (SE). While SE revealed an asymmetric but wide (>100 nm) charge depletion and accumulation layers on either side of the SSE^11^, no information about the faradaic electrochemical behavior of the SCLs could be obtained^13^. A recent EIS study revealed that the conductivity inside the SCLs is at least one order of magnitude lower than the bulk conductivity, which should significantly influence battery performance^12^. A review on the SCL formation between sulfide SSEs and oxide cathodes revealed a significant charge accumulation^16^. To understand the importance of the SCL formation in solid-state electrochemistry, a quick jump into semiconductors reveals a very insightful analogy. When two semiconductors of different chemical potential for electrons are brought into contact, a non-conductive depletion layer forms. When the same happens in between two ion conductors (such as a SSE and an electrode material), the interface resistances grows dramatically.

To rationalize the experimental results from EIS and SE by means of a theoretical model, we recently developed a simple yet predictive kinetic Monte Carlo^17,18^ (kMC) model to simulate the mass-transport phenomenon in SSEs, including the electrostatic interactions among ionic species, under blocking conditions^19^. The validity of our kMC approach was proven by reproducing the quantitative trends in SCL thicknesses and depletion layer capacitance. Moreover, the kMC simulation enabled us to determine inaccessible physical quantities via experiments such as local concentration and potential profiles as well as their time evolution into a steady state. The analysis of local concentration profiles as a function of an applied bias potential demonstrated that the depletion and accumulation layers’ perpendicular growth regime is directly connected to a fully depleted or fully occupied vacancy lattice, respectively. This observation agrees with previous experimental findings and other modeling approaches, such as thermodynamic simulations^9^. Remarkably, the kMC model requires only a minimal set of physically coherent input parameters mostly available via direct experimental measurement: (1) the bulk concentration of mobile Li-ions (${c}_{{{{{{\rm{L}}}}}}{{{{{{\rm{i}}}}}}}^{+},{{{{{\rm{bulk}}}}}}}$), (2) the maximum concentration of mobile Li-ions in a fully occupied lattice (${c}_{{{\max }}}$), (3) the relative permittivity of the bulk SSE (${\varepsilon }_{{{{{{\rm{r}}}}}}}$) and (4) the applied bias potential (${\phi }_{{{{{{\rm{bias}}}}}}}$). The consequent next step is the extension of the original setup for non-blocking conditions to investigate the mass transport between Li(s) and a corresponding oxide SSE. For this purpose, we can exploit one of the many favorable intrinsic properties of kMC: the straightforward incorporation of individual particle-based processes, such as the injection and removal of Li^+^ at the interface between metallic Li and an SSE^20^.

In the present work, the application of three methods is aimed at investigating the non-blocking conditions at the SSE/lithium metal interface in solid-state battery-relevant systems. Spectroscopic ellipsometry is used to measure the optical properties of the SSE to detect the formation of the space charge layers. Impedance spectroscopy helps to measure the ionic resistance of the SSE and formed depletion layers. Kinetic Monte Carlo simulations are used to model the mass transport processes at the interface and the transport within the SSE sample, providing kinetic information about the diffusion and migration of ions in the SSE. These methods are used together to comprehensively understand the mass transport kinetics at the SSE/lithium metal interface under non-blocking and blocking conditions.

## Results and discussion

### Proving the existence of SCLs in non-blocking conditions

**Fig. 1:**
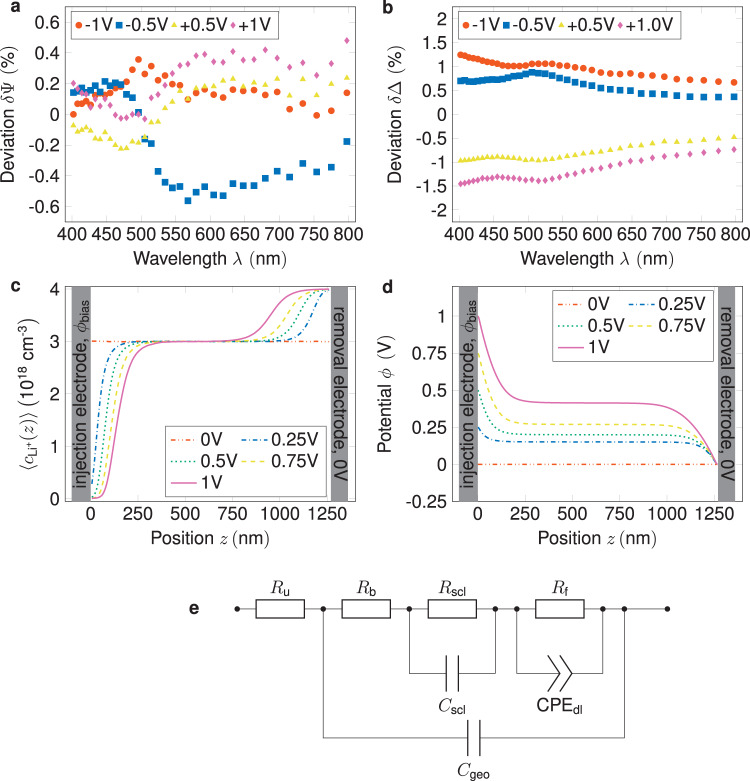
Experimental proof of the SSE phenomenon. Spectroscopic ellipsometer parameter deviations (see text) at various applied potentials. **a**, **b** are the psi and delta parameters, respectively, with similar behavior observed for both negative and positive potentials. **c** The charge distribution from the kMC simulations. It shows an asymmetric distribution of charge toward the interfaces. **d** The corresponding potential distributions from the kMC simulations, which vary depending on the applied potential. The error bars of the simulations results are smaller than the data points and, thus, omitted. **e** Equivalent electric circuit, adapted from early work, with an additional faradaic resistance to account for the mass transport across the SSE/Li(s) interface.

Proof of the existence of the much debated SCL at the Li(s)/SSE interface was the first goal of this study. In Fig. 1a, b, one can see the deviation of ellipsometry spectra when a potential is applied to the sample, as shown in Fig. 4a. The baseline spectrum (see Fig. S2 Supporting Information) was recorded under OCV conditions in our fully symmetric sample close to 0 V and subtracted from the spectra recorded under steady-state conditions with a fixed potential (−1 V, −0.5 V, +0.5 V, 1 V). Clearly, the changes in the delta parameter of the spectrum show a symmetric deviation for negative vs. positive applied potentials. Although the ellipsometry parameters ($\Delta$ and ψ) do not carry any physical meaning for such complex systems^21^, this symmetry in the deviation clearly indicates a change in the sample′s optical properties. With a clear indication of a SCL occurrence, as also seen in our previous work. The charge concentration profiles from the kMC simulations, shown in Fig. 1c indicate the presence of two distinct SCLs with the depletion layer next to the injection electrode and the accumulation layer next to the removal electrode. In Fig. 1d, the corresponding potential profiles are shown, and as expected, a constant concentration profile corresponds to a linear drop of the potential. At the end points of the simulated SSE, the potentials match the boundary conditions.

### Electrochemical properties of the SCL

The equivalent electric circuit (EEC) shown in Fig. 1e is a model used to represent the behavior of the electrochemical system. This circuit is similar to our previous model but incorporates a faradaic resistance component to account for the charge-transfer resistance under non-blocking conditions. This faradaic resistance term reflects the resistance encountered in the transfer of charge between the non-blocking electrodes and the SSE and makes it possible to explore the electrochemical behavior of the Li(s)-electrodes^22^.

**Fig. 2:**
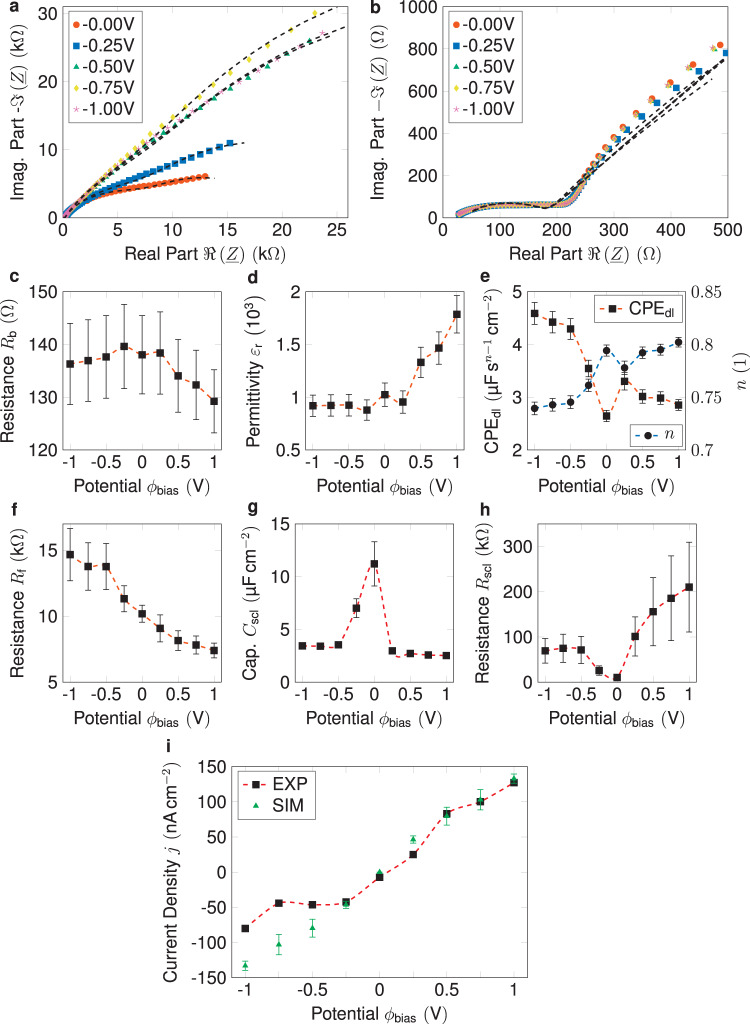
Impedance analysis and chronoamperometric measurements. **a** Full range impedance spectra of the Li/SSE/Li sample for five different positive bias potentials. Lines show a good fit for the data using the EEC shown in Fig. 1e. **b** The high-frequency region of the impedance spectra showing a good correlation between the bulk impedance and corresponding EEC fits. **c** Bulk resistance independent of applied bias potential, confirming our EEC. **d** Dielectric constant of the sample, constant for negative potentials but rising with more positive potentials. **e** Pseudocapacitance of the double layer. **f** Faradaic resistance at the Li/SSE interface decreases with increasing potential. **g** SCL capacitances symmetrically drop toward positive and negative potentials. **h** Increasing SCL resistance when a potential is applied indicates a lower ionic conductivity due to missing charge carriers. **i** Experimental and simulated current densities from chronoamperometry measurements. The error bars of the simulated current densities were obtained by block averaging over steady-state configurations. The experimental error bars show the 95% confidence intervals of the fitting algorithm.

The EIS spectra shown in Fig. 2a, b suggest that the EEC model from Fig. 1e provides a good fit for the experimental data. The Nyquist plots display the impedance spectra of the system, with the real part of impedance on the *x*-axis and the imaginary part of impedance on the *y*-axis. The fits from the EEC model are overlaid on the experimental data, demonstrating that the EEC can accurately capture the dynamic response of the system. The high-frequency region of the impedance spectra is shown in more detail in Fig. 2b, highlighting the contributions from the different components of the EEC. These results suggest that including a faradaic resistance in the EEC is essential for accurately modeling the charge-transfer resistance in non-blocking conditions. The EIS spectra for the equivalent range of positive bias potentials are presented in Fig. S3 Supporting Information.

As illustrated in Fig. 2c, the bulk resistance remains almost constant despite a 10% relative estimated error. Since the bias potential affects only the interface properties, this observation further validates the EEC. On the other hand, the dielectric permittivity in Fig. 2d, which relies heavily on the number of mobile Li-ions in the SSE, changes when a net positive current is applied and is determined based on the geometric capacitance. Repeated experiments indicate no hysteresis, suggesting that the change in dielectric properties is not due to any irreversible chemical degradation of the SSE but rather to the varying concentration of mobile Li-ions within the SSE.

As explained in more detail within our previous work, the ionic charge accumulation in the form of the SCLs is accompanied by a dense and thin double-layer (DL), like the Helmholtz layer found in liquid electrolytes^23^. The pseudocapacitance value of this DL is shown in Fig. 2e and varies between 2–5 µF s^1 − *n*^ cm^−2^ with an *n*-value between 0.75 and 0.85.

Overall, the impedance data reveals a similar pattern to the blocking conditions. The SCL capacitance, which can later be used to estimate the SCL thickness and compared with other measurements, is found to be four times lower than observed under blocking conditions, but the qualitative trend remains the same. Figure 2i shows the chronoamperometry data of the sample that has undergone impedance analysis. From an electrochemical perspective, a straight line would be a reasonable outcome for this type of measurement, confirming a perfectly ohmic behavior of the electrodes. However, slight deviations from this behavior can be observed for very low potentials below −0.5 V, which can be explained by the electrochemical changes to the electrode. The faradaic resistance, *R*_f_, shown in Fig. 2f, is in good agreement with this deviation. The simulated data in Fig. 2i is based on the values for the injection and removal rates of the kMC model. The space-charge properties ${R}_{{{{{{\rm{scl}}}}}}}$ and ${C}_{{{{{{\rm{scl}}}}}}}$, in Fig. 2g, h show the typical symmetric behavior in dependence of the applied potential, where only a thin SCL is formed at 0 V bias potential. Thus, no significant resistance is present and the capacity is high due to the thin layer. As outlined above, the injection and removal rates of the kMC model were parametrized to match the experimental results. The experimental deviation can be explained through the non-ohmic nature of the Li(s) electrodes, a commonly observed behavior in the literature^24^. The remaining EIS parameters ${C}_{{{{{{\rm{geo}}}}}}}$ and ${R}_{{{{{{\rm{u}}}}}}}$ are shown in Fig. S1 Supporting Information.

### Influence of mass transport over the Li(s)/SSE interface

The modeling of mass transport over the interface as an energy-independent process is undoubtedly a simplified concept. Nevertheless, the kMC model enables us to draw important conclusions regarding the main system dynamics. Comparing the injection/removal rates with the maximum transport rate shows that mass transport within the SSE is by a factor of ${10}^{7}$ faster than mass transport through the Li(s)/SSE interface. Consequently, the actual SCL formation is temporally decoupled from the mass transport over the electrodes. Upon application of bias potential, an accumulation and a depletion layer form rapidly at the respective contacts. A completely depleted and full vacancy lattice at the corresponding Li(s)/SSE interface generates a favorable occupation situation for Li^+^-injection and Li^+^-removal, respectively. From a kinetic point of view, the kMC model indicates that mass transport through the Li(s)/SSE interface is (1) a symmetric phenomenon (equal injection/removal rates) and (2) such slow that its influence on SCL formation is in fact negligible. These findings agree with the experimental observation that the SCL formation under blocking and non-blocking conditions yields similar results. On the other hand, parametrization of the simulation model with strongly asymmetric injection/removal rates eventually would lead to the formation of either two accumulation or two depletion layers, which experiments cannot observe.

### Unifying comparison of experiments and simulations

**Fig. 3:**
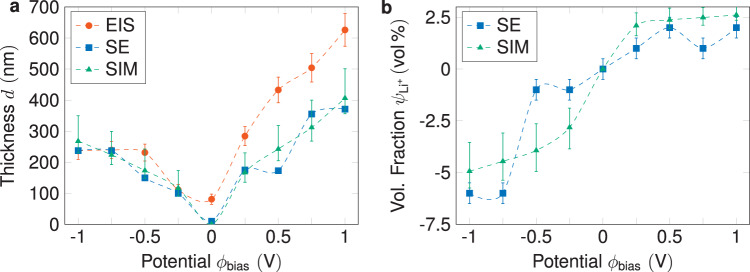
Comparison of experimental and simulation results. **a** Comparison of SCL thicknesses calculated from different methods. The thicknesses calculated from EIS, SE, and kMC simulations for different applied potentials. It demonstrates that the thicknesses calculated from all three methods are in good agreement with the different applied potentials. This confirms the consistency and reliability of the results obtained from the different techniques used in this study. **b** Volume fraction change in vol% based on the fit of the SE data and the kMC simulations, based on a Li-ion density of ${4.5\times 10}^{21}{{{{{{\rm{cm}}}}}}}^{-3}$, including mobile and immobile ions. The error bars of the kMC simulation were calculated based on the resolution accuracy of the thicknesses determined by SE. The experimental error bars show the 95% confidence intervals of the fitting algorithm.

The consistency of the different approaches, which, except for the feedback loops from the experimentally determined current densities to the injection and removal rates of the kMC electrodes, are completely independent of one another, can be seen in Fig. 3a. In order to understand the correlations between the three methods, the electrochemical property of a charged layer near the interface can be explained as follows: a region of lower Li-ion concentration such as the SCL is equivalent to an SSE with lower conductivity, which leads to an increase of resistance in the impedance. The charge accumulation is proportional to the SCL thickness, as the model suggests a perpendicular growth into the SSE. The thickness of the SCLs, all in the range of 100–600 nm and asymmetrically rising with increasing potentials, are consistent within the three techniques. The overestimation of the SCL thicknesses at positive potentials, can be explained by the way it is calculated from the impedance data. The geometric capacitance (see Fig. S1a Supporting Information) is used to calculate the dielectric constant of the (bulk) SSE, which is then used to calculate the thickness from the SCL capacitance. Herein, we assume that the concentration of Li-ions does not change the dielectric constant of the SSE, which is clearly not true for larger concentration changes, as seen in Fig. 2d. Finally, SE also enables us to extract the fraction change of Li-content, ${\psi }_{{{{{{\rm{L}}}}}}{{{{{{\rm{i}}}}}}}^{+}}$, with respect to the bulk concentration in vol%, see Fig. 3b. Negative and positive concentration changes are another indicator of the existence of a depletion and accumulation layer. A direct comparison of ${\psi }_{{{{{{\rm{L}}}}}}{{{{{{\rm{i}}}}}}}^{+}}$ with the results from the kMC model is not possible as the kMC model only considers mobile Li^+^, and the volume fraction change is calculated with respect to the total bulk concentration, that is, mobile and immobile Li-ions. However, we may perform an indirect comparison by adopting a fixed total bulk concentration for the kMC model. In Fig. 3b, we obtain a decent match with the experimental profile by assuming a total Li-ion density of ${4.5\times 10}^{21}\,{{{{{{\rm{cm}}}}}}}^{-3}$ to compute a corresponding profile from the simulation data. The given total bulk concentration is by a factor of 1500 larger than the bulk concentration of mobile Li^+^ used in the kMC model, which is in good agreement with values from pertinent literature^23^.

## Conclusions

Spectroscopic ellipsometry allows for direct measurement of the SCL thicknesses for different applied bias potentials. With the occurrence of a highly resistive layer in the SSE upon application of a potential in our sample, a deeper look into its properties is used to shed light on the size and Li-ion concentration change. With its occurrence proven by SE, the electrochemical properties are tested through electrochemical impedance spectroscopy. Finally, the parameterized kMC model is shown to have large predictive power and can be used in the future to assess the impact of ionic charge accumulation at the interface of a newly developed anode and solid-state electrolytes.

Despite the controversies in existing literature, the occurrence of SCLs is reliably and reproducibly shown by three different methods, wherein each method has its own unique capability to characterize the SCL. Importantly, the consistency of the approaches is shown with single parameters that can be very easily compared.

The nature of these highly charged layers can explain the widely known degradation at the interface between Li(s) anodes and the SSEs and therefore lay the foundation for a better understanding of how to prevent this instability. Once the interface can be engineered by tuning the materials or creating an interfacial layer^25^ to prevent such SCL formation, this can greatly benefit the enabling of all-solid-state batteries with Li(s) anodes.

## Methods

### Experimental and simulation setups

**Fig. 4:**
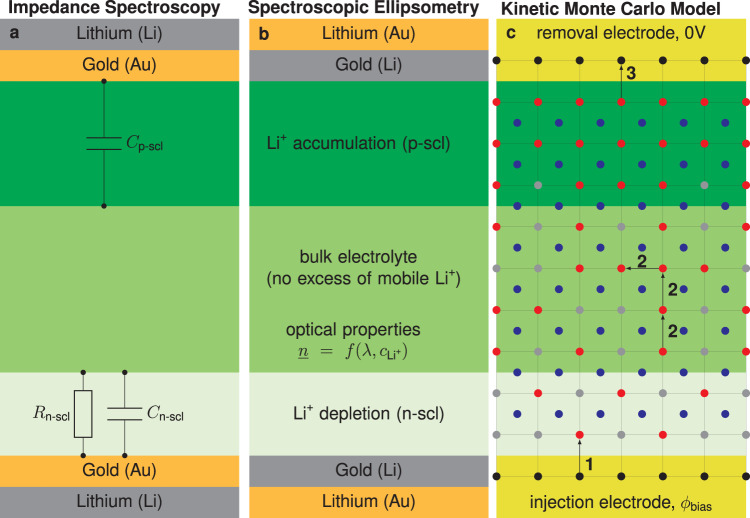
Experimental setups and kinetic Monte Carlo (kMC) model. Schematic representation of (**a**) the spectroscopic ellipsometry (SE) and (**b**) the electrochemical impedance spectroscopy (EIS) setups used in the experiments. **c** Schematic representation of the kMC model used to simulate the behavior of charge accumulation at the interface between a lithium metal electrode and an oxide solid-state electrolyte. Gray, red, and blue dots represent unoccupied lattice vacancies, mobile Li^+^-ions and their immobile counteranions, respectively. The metallic Li-electrodes are illustrated by black dots and act as source and sink for mass-transport. The numbers correspond to the three implemented dynamic transitions: (1) Li^+^-injection, (2) Li^+^-transport and (3) Li^+^-removal.

In Fig. 4, the different measurement setups are shown, which were used to perform the two experimental techniques (SE in Fig. 4a, EIS in Fig. 4b) and a sketch of the kMC model in Fig. 4c. The experimental design was carefully chosen to prevent a tandem of instabilities from interfering with the measurement: (1) the reduction of the Li(s) when in contact with air, (2) the reaction of the SSE when in touch with Li(s)^26^. As the SE measurements are relatively fast (multiple hours) but are done under ambient conditions, the Au-layer on top of the Li(s) electrode provides protection from the atmosphere. On the other hand, the EIS measurements are relatively slow but can be performed in an argon atmosphere, and the Au-layer between Li(s) and SSE acts as a passivation layer between the two materials^27^. More details on the preparation and conditions of the measurement can be found in the experimental section.

Next, we outline the extended model setup for an SSE sample contacted by two metallic Li-electrodes, see Fig. 4c. Here, we briefly summarize the most important aspects of the original model^19^. The device is mapped to a three-dimensional Cartesian lattice of volume $V=X\times Y\times Z=31.5\times 31.5\times 1260\,{{{{{{\rm{nm}}}}}}}^{3}$ with a lattice constant of ${a}_{{{{{{\rm{L}}}}}}}=6.3\,{{{{{\rm{nm}}}}}}$ and periodic boundary conditions in the ${xy}$-plane. The bottom and top layer in $z$-direction correspond to the Li(s)-electrodes which either act as a sink or source for Li-Ions (in the following denoted as removal and injection electrode, respectively). Note that the model does not distinguish between the Au-layer and the Li(s) electrode but instead treats them as an ideal contact with ${\varepsilon }_{{{{{{\rm{r}}}}}}}\to \infty$. The region confined between the contacts models the SSE sample where each node $i$ represents an unoccupied vacancy site. The sample is populated with mobile Li-ions according to a particular bulk concentration ${c}_{{{{{{\rm{L}}}}}}{{{{{{\rm{i}}}}}}}^{+},{{{{{\rm{bulk}}}}}}}$. The value of ${c}_{{{{{{\rm{L}}}}}}{{{{{{\rm{i}}}}}}}^{+},{{{{{\rm{bulk}}}}}}}$ was recently assessed in the scope of an ionic Mott-Schottky formalism to be in the range of ${2-4\times 10}^{18}\,{{{{{{\rm{cm}}}}}}}^{-3}$^28^. In the scope of this work, we utilize the mean value ${c}_{{{{{{\rm{L}}}}}}{{{{{{\rm{i}}}}}}}^{+},{{{{{\rm{bulk}}}}}}}={3\times 10}^{18}\,{{{{{{\rm{cm}}}}}}}^{-3}$ as a first order approximation. In general, the concentration of mobile cations, ${c}_{{{{{{\rm{L}}}}}}{{{{{{\rm{i}}}}}}}^{+}}$, and its physical boundaries play a key role in the asymmetric SCL formation in SSEs. Recently^19^, we established that:

1 $${c}_{{{\min }}}\le {c}_{{{{{{\rm{L}}}}}}{{{{{{\rm{i}}}}}}}^{+}}\le {c}_{{{\max }}},$$

where ${c}_{{{\min }}}$ and ${c}_{{{\max }}}$ denote the minimum and maximum concentration of Li^+^ in a fully depleted and fully occupied lattice, respectively. In the present model, we naturally set ${c}_{{{\min }}}=0,$ whereas as the inverse volume of a unit cell imposes the maximum concentration, ${c}_{{{\max }}}={a}_{{{{{{\rm{L}}}}}}}^{-3}$. A homogeneously distributed anionic background is implemented to ensure electroneutrality with respect to the sample′s initial condition. The presence of immobile Li-ions^29^ is neglected as corresponding counter anions locally neutralize them and thus do not alter the underlying energetic landscape for the transport of Li^+^. Analogously to liquid electrolytes^30^, the strength of electrostatic screening also impacts the thicknesses of the resulting SCLs. Here, we control the magnitude of this effect via the relative permittivity ${\varepsilon }_{{{{{{\rm{r}}}}}}}$ of the bulk SSE.

### Modeling of Li-ion dynamics

Our model features three types of dynamic transitions (cf. numbers in Fig. 4c):

1. Li^+^-injection from the source electrode.
2. Li^+^-transport guided by a thermally activated hopping mechanism^31,32^.
3. Li^+^-removal from the sink electrode.

Li-ions can move to unoccupied nearest neighbor’s vacancies via hopping transport which is affected by the local values of the potential energy surface ${E}_{i}$. These local energy levels comprise three different energetic contributions: the energy defined by a reference electrode ${E}_{i}^{{{{{{\rm{ref}}}}}}}$, the contribution from an external electric field ${E}_{i}^{{{{{{\rm{F}}}}}}}$ and the influence of Coulomb interactions of mobile cations and their respective immobile counter anions. In summary, the total potential energy at vacancy site $i$ is given by:

2 $${E}_{i}={E}_{i}^{{{{{{\rm{ref}}}}}}}+{E}_{i}^{{{{{{\rm{F}}}}}}}+{E}_{i}^{{{{{{\rm{C}}}}}}}.$$

In the present study, we only consider energy differences ${\Delta E}_{{ij}}$ between two vacancy sites $i$ and $j$ and, thus, we may set ${E}_{i}^{{{{{{\rm{ref}}}}}}}=0$. ${E}_{i}^{{{{{{\rm{F}}}}}}}$ is assumed to drop linearly in $z$-direction across the contacted SSE sample, that is:

3 $${E}_{i}^{{{{{{\rm{F}}}}}}}=\left(q{\phi }_{{{{{{\rm{b}}}}}}}-\Delta W\right)\frac{{z}_{i}}{Z}$$

where ${\phi }_{{{{{{\rm{b}}}}}}}$ denotes the applied bias potential, $\Delta W$ is the difference in electrode work functions and ${z}_{i}$ is the $z$-coordinate of the site $i$. For identical electrodes, we may set $\Delta W=0$. While the first two contributions are held constant during the simulation, ${E}_{i}^{{{{{{\rm{C}}}}}}}$ must be updated dynamically. The model considers the interaction of mobile cations (cation–cation interactions), ${E}_{i}^{{{{{{\rm{cc}}}}}}}$, and interaction of mobile cations with immobile counteranions (cation–anion interaction) ${E}_{i}^{{{{{{\rm{ac}}}}}}}$. Both contributions are computed accurately via a three-dimensional Ewald summation adjusted for a contacted infinite slab-device as established by Casalegno et al.^33,34^. Due to the fixed positions of anions, the values of ${E}_{i}^{{{{{{\rm{ac}}}}}}}$ can be calculated before the simulation and cached on related vacancy sites. On the other hand, ${E}_{i}^{{{{{{\rm{cc}}}}}}}$ depends on the current spatial distribution of all mobile cations and must be updated accordingly in each kMC step. In the context of Coulomb interactions, special attention must be paid to non-electroneutral device configurations as they can lead to convergency issues^35^. Under non-blocking conditions, such arrangements could arise from strongly asymmetric injection and removal rates. However, please note that the applied electrostatic solver implicitly handles such cases by extending the original simulation box with a corresponding box of image charges representing the polarization of an ideal metal contact. To reduce the computational effort arising from the dynamic calculation of Coulomb interactions, we apply a combination of different strategies^19^, particularly the so-called dipole-update method^36^.

The thermally activated hopping of cations between vacancies sites $i\to j$ is captured via the Miller–Abrahams formula^37^:

4 $${k}_{ij}={k}_0\cdot \left\{\begin{array}{c}{{{{{\rm{exp }}}}}}\left(-\frac{\Delta {E}_{{ij}}}{{E}_{{{{{{\rm{th}}}}}}}}\right),\quad\Delta {E}_{{ij}} \, < \, 0\\ \quad\quad\quad\quad1,\quad\Delta {E}_{{ij}}\ge 0\end{array},\right.$$

where ${k}_{0}$ is the attempt-to-hop frequency, ${\Delta E}_{{ij}}$ denotes the difference in potential energy between vacancy $i$ and $j$ and ${E}_{{{{{{\rm{th}}}}}}}={k}_{{{{{{\rm{B}}}}}}}T$ is the thermal energy. The attempt-to-hop frequency is estimated from an Arrhenius equation^38^:

5 $${k}_{0}=\frac{{k}_{0,{{\max }}}}{{a}_{{{{{{\rm{L}}}}}}}^{2}}{{\exp }}\left(-\frac{{E}_{{{{{{\rm{a}}}}}}}}{{E}_{{{{{{\rm{th}}}}}}}}\right),$$

where ${k}_{0,{{\max }}}={E}_{{{{{{\rm{th}}}}}}}/h$ and ${E}_{{{{{{\rm{a}}}}}}}$ denotes an experimentally 5obtained activation energy for diffusion^39^. We scale ${k}_{0,{{\max }}}$ by ${a}_{{{{{{\rm{L}}}}}}}^{-2}$ similarly to a three-dimensional random walk based on the Einstein-Smoluchowski treatment for Brownian motion^40^. When Li-ions reside on vacancy sites neighboring to contact nodes, they can be removed from the SSE sample with a constant rate ${k}_{{{{{{\rm{rem}}}}}}}$. Therefore, the cumulative removal rate is given by:

6 $${K}_{{{{{{\rm{rem}}}}}}}={n}_{{{{{{\rm{L}}}}}}{{{{{{\rm{i}}}}}}}^{+},{{{{{\rm{contact}}}}}}}{k}_{{{{{{\rm{rem}}}}}}}$$

where ${n}_{{{{{{\rm{L}}}}}}{{{{{{\rm{i}}}}}}}^{+},{{{{{\rm{contact}}}}}}}$ is the total number if Li-ions residing next to the contact. Vice versa, Li^+^ can be injected into an unoccupied vacancy site from the contact with the rate ${k}_{{{{{{\rm{inj}}}}}}}$ and, accordingly, the cumulative injection rate is given by:

7 $${K}_{{{{{{\rm{inj}}}}}}}=\left({n}_{{{{{{\rm{contact}}}}}}}-{n}_{{{{{{\rm{L}}}}}}{{{{{{\rm{i}}}}}}}^{+},{{{{{\rm{contact}}}}}}}\right){k}_{{{{{{\rm{inj}}}}}}}$$

where ${n}_{{{{{{\rm{contact}}}}}}}$ denotes the total number of contact sites.

### Experimental section, data evaluation and model parametrization

#### Solid-state electrolyte

LICGC^**TM**^ (Ohara Inc, Japan) was used for electrochemical and optical experiments conducted in this study. The SSE had a thickness of 150 µm and was stable in the ambient atmosphere.

#### Gold/lithium electrodes

All electrode depositions were performed in an argon glovebox with a highly inert atmosphere (O_2_ < 0.1 ppm, H_2_O < 0.1 ppm). Au electrodes were thermally evaporated symmetrically using a MICO evaporator (Tectra, Germany) with an evaporation rate of 1 Å s^−1^ and a final thickness of 25 nm. The Li electrodes were evaporated under the same conditions. The order of deposition was chosen to match the desired sample structures for EIS and SE measurements.

#### Spectroscopic ellipsometry

An EP4 imaging ellipsometer (Accurion, Germany) was used to perform spectroscopic ellipsometry at different potentials, and in situ ellipsometry was done at an angle of incidence (AOI) of 65° using a 658 nm solid-state laser. For spectroscopic measurements, the wavelength from 360 to 1000 nm in 50 equidistant energy steps was adjusted using a built-in grading monochromator and a laser-stabilized xenon arc lamp. A resting period of 2.5 h after applying the bias potential and before the spectroscopic scans was used to allow the system to reach electrochemical equilibrium.

#### Electrochemical impedance spectroscopy

The AC impedance measurements were carried out with a VSP300 potentiostat (Biologic, France) in the frequency range between 3 MHz and 3 Hz with a probing signal amplitude of 10 mV. The metal-contacted samples were assembled into a PAT-Cell (EL-CELL, Germany) with polished stainless-steel plungers to contact the electrode area. The cells were placed into a PAT-Stand (EL-CELL, Germany) with a 3 m cable to the potentiostat. The impedance of the samples was measured in the bias range between −1.0 V and +1.0 V (vs. EOC, EOC = +0.11 V). After applying the bias potential, a waiting time of 15 min was used to ensure electrochemical equilibrium. The impedance data were analyzed using the “EIS Data Analysis 1.3” software^41^.

#### Kinetic Monte Carlo simulations

A single run of the kMC model produces one possible many-body time evolution of the investigated device into its steady state. By block-averaging over steady-state configurations^33^, we obtain three-dimensional concentration and potential profiles denoted as ${c}_{{{{{{{\rm{Li}}}}}}}^{+}}\left(x,y,z\right)$ and $\phi \left(x,y,z\right)$, respectively. The potential profiles are directly computed via the underlying electrostatic solver, as outlined above. As our device model does not contain any local structural or energetic inhomogeneities, all three-dimensional profiles are homogeneous within the ${xy}$-plane. Thus, we may compute averaged profiles, $\left\langle {c}_{{{{{{{\rm{Li}}}}}}}^{+}}\left(z\right)\right\rangle$ and $\left\langle \phi \left(z\right)\right\rangle$, to facilitate visualization and further rationalization. To compare the simulation outputs with data from EIS and SE, we extracted the thicknesses of the accumulation and depletion layer, denoted as ${d}_{{{{{{\rm{p}}}}}}-{{{{{\rm{scl}}}}}}}$ and ${d}_{{{{{{\rm{n}}}}}}-{{{{{\rm{scl}}}}}}}$, respectively. The average values of both layers as a function of ${\phi }_{{{{{{\rm{bias}}}}}}}$ are determined from $\left\langle {c}_{{{{{{{\rm{Li}}}}}}}^{+}}\left(z\right)\right\rangle$ via the criteria $\left\langle {c}_{{{{{{{\rm{Li}}}}}}}^{+}}\left(z\right)\right\rangle \,\le \left(1-\delta \right)\cdot {c}_{{{{{{\rm{L}}}}}}{{{{{{\rm{i}}}}}}}^{+},{{{{{\rm{bulk}}}}}}}$ and $\left\langle {c}_{{{{{{{\rm{Li}}}}}}}^{+}}\left(z\right)\right\rangle \,\ge \left(1+\delta \right)\cdot {c}_{{{{{{\rm{L}}}}}}{{{{{{\rm{i}}}}}}}^{+},{{{{{\rm{bulk}}}}}}}$ for ${d}_{{{{{{\rm{n}}}}}}-{{{{{\rm{scl}}}}}}}$ and ${d}_{{{{{{\rm{p}}}}}}-{{{{{\rm{scl}}}}}}}$, respectively, where $\delta$ corresponds to the resolution accuracy of the thicknesses determined by SE. In the present work, we set $\delta =0.05$. Upper and lower boundaries for ${d}_{{{{{{\rm{p}}}}}}-{{{{{\rm{scl}}}}}}}$ and ${d}_{{{{{{\rm{n}}}}}}-{{{{{\rm{scl}}}}}}}$ are computed via the above criteria by setting $\delta =0.01$ and $\delta =0.1$. Finally, the kMC model also enables us to evaluate the current density over the injection and removal electrode:

8 $${j}_{{{{{{\rm{inj}}}}}}/{{{{{\rm{rem}}}}}}}=\frac{q{N}_{{{{{{\rm{inj}}}}}}/{{{{{\rm{rem}}}}}}}}{A\Delta t}$$

where $\Delta t$ is the total simulated time, *A* is the electrode area in the ${xy}$-plane, $q$ is the elementary charge, and ${N}_{{{{{{\rm{inj}}}}}}/{{{{{\rm{rem}}}}}}}$ is the number of injection/removal events in $\Delta t$. In a steady-state configuration ${j}_{{{{{{\rm{inj}}}}}}}\approx {j}_{{{{{{\rm{rem}}}}}}}$ holds so that the stationary current density over the device is just denoted as $j$. The statistical errors of $j$ are also determined via block-averaging over steady-state configurations, as mentioned above. An overview of all symbols utilized in the present study is given in Table S1 Supporting Information.

The parameterization of the kMC model is exclusively based on the experimentally obtained results. Since the kMC setup is symmetric and allows for the extraction of the depletion and accumulation layer, we only simulate positive bias potentials ${\phi }_{{{{{{\rm{bias}}}}}}}$ from 0 V to 1 V in steps of 0.25 V. The bulk concentration is set to ${c}_{{{{{{\rm{L}}}}}}{{{{{{\rm{i}}}}}}}^{+},{{{{{\rm{bulk}}}}}}}={3\times 10}^{18}\,{{{{{{\rm{cm}}}}}}}^{-3}$ according to the above-mentioned ionic Mott-Schottky formalism^23^. The maximum concentration is limited to ${c}_{{{\max }}}=\frac{4}{3}{c}_{{{{{{\rm{L}}}}}}{{{{{{\rm{i}}}}}}}^{+},{{{{{\rm{bulk}}}}}}}={4\times 10}^{18}\,{{{{{{\rm{cm}}}}}}}^{-3}$ based on the ratio of change in Li^+^ concentration obtained from SE, see Fig. 4b. The relative permittivity of the bulk SSE ${\varepsilon }_{{{{{{\rm{r}}}}}}}$ is varied with increasing ${\phi }_{{{{{{\rm{bias}}}}}}}$ in accordance with the results from EIS for the geometric capacitance (cf. Fig. 2d). Furthermore, all experimental results indicate that the device remains approximately charge-neutral even for non-blocking conditions. Thus, we may set ${k}_{{{{{{\rm{inj}}}}}}}={k}_{{{{{{\rm{rem}}}}}}}$ as disparate rates for injection and removal of Li^+^ from the electrodes induce a device state which deviates from charge-neutrality. We parametrize the values for ${k}_{{{{{{\rm{inj}}}}}}}$ and ${k}_{{{{{{\rm{rem}}}}}}}$ to reproduce the current densities obtained from chronoamperometric measurements. A summary of all potential-dependent input parameters is given in Table 1.

**Table 1:** Parametrization of the kinetic Monte Carlo model.

${\phi }_{{{{{{\rm{bias}}}}}}}$ (V)	0	0.25	0.50	0.75	1
${\varepsilon }_{{{{{{\rm{r}}}}}}}$ (1)	900	1000	1150	1350	1700
${k}_{{{{{{\rm{inj}}}}}}}/{k}_{{{{{{\rm{rem}}}}}}}$ (s^−1^)	0.0	0.0625	0.1875	0.25	0.3125

Summary of potential-dependent input parameters of the kMC model.

## Supplementary information

Supplemental Information

## Data Availability

All relevant data are available from the authors upon reasonable request. Any request can be addressed to A.S.B. for experimental data and A.G. for simulation data.

## Code availability

The simulation code of the space-charge layer formation in solid-state electrolytes for (non-) blocking conditions has been implemented within C++ in our in-house kinetic Monte Carlo framework^42^. The source code is not yet openly accessible as it is part of a larger software project that will be published separately later. Each kMC simulation was run on eight cores of an AMD Ryzen^TM^ Threadripper^TM^ 3990X @2.9 GHz with 64 hardware cores. Per fixed input parameter combination, the simulation time to obtain a simulated time of 50 s averaged out at ~7 days.
