# Supplementary material for: Mass transport and charge transfer through an electrified interface between metallic lithium and solid-state electrolytes
Source: Commun Chem. 2023 Jun 15;6:124. doi: 10.1038/s42004-023-00923-4 (PMC10272140; doi:10.1038/s42004-023-00923-4)
Supplement: Supplementary file 1 — Supplemental Information [file 42004_2023_923_MOESM1_ESM.pdf]

SUPPORTING INFORMATION:

Mass Transport and Charge Transfer through an Electrified Interface between Metallic Lithium and Solid-State Electrolytes

Leon Katzenmeier,[a,b,1] *Manuel Gößwein,*[c,1]

Leif Carstensen,[a,b] *Johannes Sterzinger,*[b] *Michael Ederer,*[b]

Peter Müller-Buschbaum,[d,e] *Alessio Gagliardi,*[c,]* *Aliaksandr S. Bandarenka*[a,]*

(1) These authors contributed equally.

[a] Technical University of Munich, TUM School of Natural Sciences, Department of Physics, Physics of Energy Conversion and Storage, James-Franck-Str. 1, 85748 Garching, Germany

[b] TUMint·Energy Research, Lichtenbergstr. 4, 85748 Garching bei München, Germany

[c] Technical University of Munich, TUM School of Computation, Information and Technology Department of Electrical and Computer Engineering, Hans-Piloty-Straße 1, 85748 Garching bei München, Germany

[d] Technical University of Munich, TUM School of Natural Sciences, Department of Physics, Chair for Functional Materials, James-Franck-Str. 1, 85748 Garching, Germany

[e] Heinz Maier-Leibnitz Zentrum (MLZ), Technical University of Munich, Lichtenbergstr. 1, 85748 Garching, Germany

*E-mail: alessio.gagliardi@tum.de; bandarenka@ph.tum.de

Tab. S1. List of Symbols

A	Electrode area in xy-plane, see Eq. 8
a_L	Lattice constant of three-dimensional Cartesian simulation grid
C_geo	Geometrical capacitance, see Fig. 1e
C_scl	Space charge layer capacitance, see Fig. 1e and Fig. 2g
$\mathrm{CPE_{dl}}$	Pseudocapacitance of double layer, see Fig. 1e and Fig. 2e
$c_{\mathrm{Li^+,bulk}}$	Bulk concentration of mobile Li-ions, see Eq. 1
$c_{\mathrm{Li^+}}(x,y,z)$	Three-dimensional concentration profile of mobile Li-ions
$\langle c_{\mathrm{Li^+}}(z)\rangle$	Spatially averaged concentration profile of mobile Li-ions, see Fig. 1c
c_max	Maximum concentration of mobile Li-ions, see Eq. 1
c_min	Minimum concentration of mobile Li-ions, see Eq. 1
Δ	Phase difference in SE
ΔE_{ij}	Difference in potential energy between vacancy i and j, see Eq. 4
ΔW	Difference in electrode work functions, see Eq. 3
Δt	Total simulated time, see Eq. 8
δ	Resolution accuracy of SCL thicknesses determined by SE
$\delta\Delta$	Relative deviation in phase difference, see Fig. 1c
$\delta\Psi$	Relative deviation in amplitude component, see Fig. 1b
d	Thickness of space charge layers, see Fig. 3a
$d_{\mathrm{p-scl}}$	Thickness of accumulation layer
$d_{\mathrm{n-scl}}$	Thickness of depletion layer
ε_r	Relative permittivity of bulk SSE, see Tab. 1 and Fig. 2d
E_a	Experimentally determined activation energy for cation diffusion, see Eq. 5
E_i	Local value of potential energy surface at site i, see Eq. 2
E_i^C	Coulomb potential at site i, see Eq. 2
E_i^F	Electric field contribution to potential energy at site i, see Eq. 2 and Eq. 3
E_i^ac	Coulomb interaction of mobile cations with immobile counteranions at site i
E_i^cc	Coloumb interaction of mobile cations at site i
E_i^ref	Energy defined by reference electrode at site i, see Eq. 2
E_th	Thermal energy, see Eq. 4 and Eq. 5
j	Steady-state current density over injection/removal electrode
j_inj	Current density over injection electrode, see Eq. 8
j_rem	Current density over removal electrode, see Eq. 8
K_inj	Cumulative cation injection rate, see Eq. 7
K_rem	Cumulative cation removal rate, see Eq. 6
k_0	Attempt-to-hop frequency for cation hopping, see Eq. 4 and Eq. 5
$k_{0,\mathrm{max}}$	Maximum frequency for cation hopping, see Eq. 5
k_{ij}	Local cation hopping rate between vacancy sites $i \rightarrow j$, see Eq. 4
k_inj	Constant cation injection rate, see Eq. 7 and Tab. 1

k_{rem}	Constant cation removal rate, see Eq. 6 and Tab. 1
λ	Optical wavelength in SE, see Fig. 1a and Fig. 1b
N_{inj}	Number of injection events in simulated time Δt, see Eq. 8
N_{rem}	Number of removal events in simulated time Δt, see Eq. 8
n_{contact}	Total number of contact sites, see Eq. 7
$n_{\mathrm{Li^+,contact}}$	Number of mobile Li-ions next to the removal electrode, see Eq. 6 and Eq. 7
q	Elementary charge, see Eq. 3 and Eq. 8
R_{b}	Bulk resistance, see Fig. 1e and Fig. 2c
R_{f}	Faradaic resistance at Li/SSe interface, see Fig. 1e and Fig. 2f
R_{scl}	Space charge layer resistance, see Fig. 1e and Fig. 2h
R_{u}	Uncompensated resistance, see Fig. 1e
ϕ_{bias}	Applied bias potential, see Tab. 1
$\phi(x,y,z)$	Three-dimensional potential profile
$\langle\phi(z)\rangle$	Spatially averaged potential profile, see Fig. 1d
Ψ	Amplitude component in SE
$\psi_{\mathrm{Li^+}}$	Volume fraction change of Li-content with respect to bulk concentration, see Fig. 3b
V	Volume of simulation grid
X	Dimension of simulation grid in x-direction
Y	Dimension of simulation grid in y-direction
Z	Dimension of simulation grid in z-direction
z_i	z-coordinate of site i, see Eq. 3

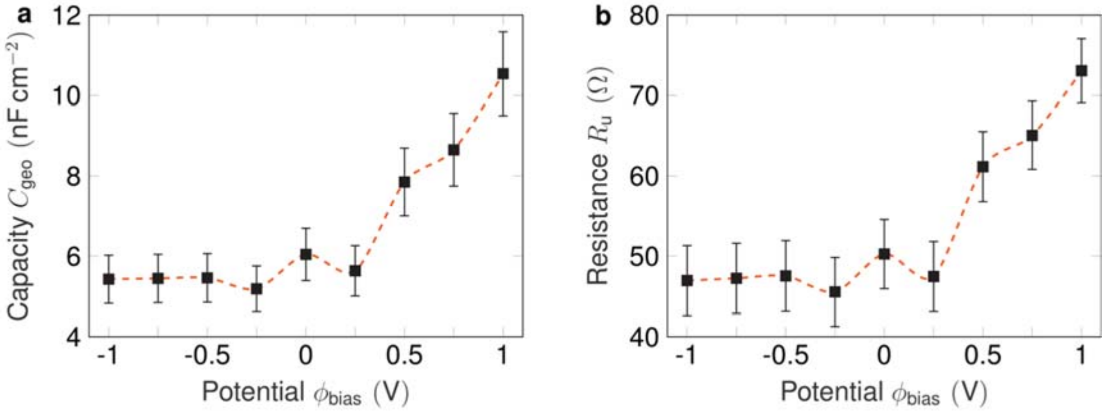

Fig. S1 Further EIS parameters based on the EEC of the main manuscript. **a** The geometric capacity of the sample in the order of nF cm^{-2}. **b** The uncompensated resistance of the sample in the sample holder.

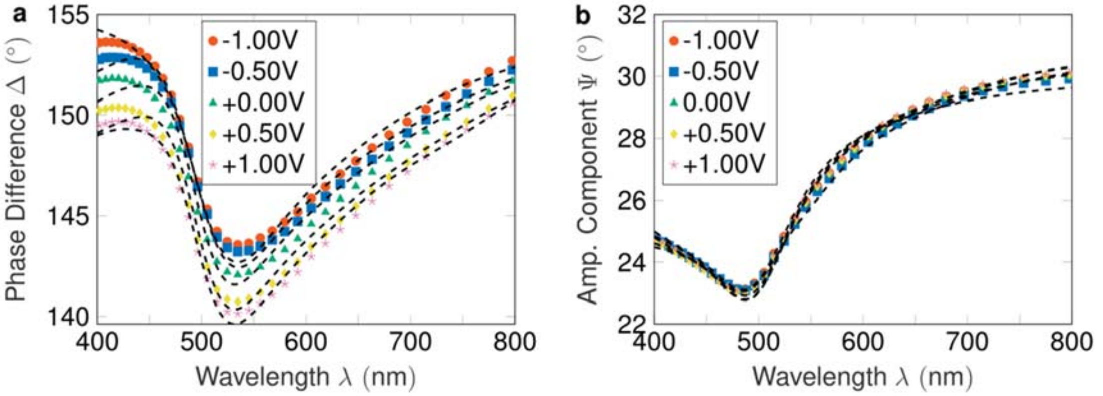

Fig. S2 Spectroscopic ellipsometry angles delta and phi for all applied potentials. a The Phase difference. **b** The Amplitude Component.

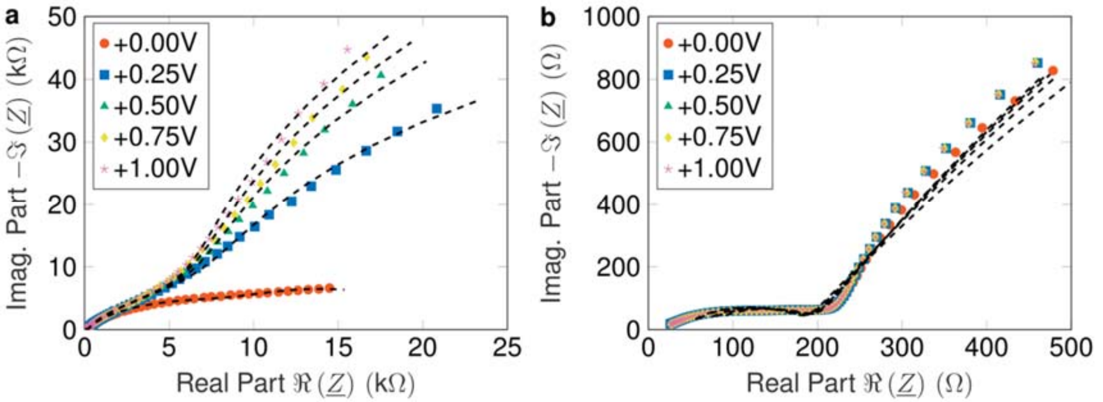

Fig. S3 Impedance spectra in a Nyquist representation. a Full frequency range. **b** Zoom into the high frequency range, revealing the bulk contribution in the form of a semicircle.
